# The effect of oxytocin on attention to angry and happy faces in chronic depression

**DOI:** 10.1186/s12888-016-0794-9

**Published:** 2016-04-06

**Authors:** Gregor Domes, Claus Normann, Markus Heinrichs

**Affiliations:** Department of Psychology, Laboratory for Biological and Personality Psychology, University of Freiburg, Stefan-Meier-Strasse 8, D-79104 Freiburg, Germany; Freiburg Brain Imaging, University Medical Center Freiburg, Freiburg, Germany; Department of Psychiatry and Psychotherapy, University Medical Center Freiburg, Freiburg, Germany

**Keywords:** Oxytocin, Chronic depression, Facial emotions, Attention, Dot probe, Social perception

## Abstract

**Background:**

Chronic depression is characterized by a high degree of early life trauma, psychosocial impairment, and deficits in social cognition. Undisturbed recognition and processing of facial emotions are basic prerequisites for smooth social interactions. Intranasal application of the neuropeptide oxytocin has been reported to enhance emotion recognition in neuropsychiatric disorders and healthy individuals. We therefore investigated whether oxytocin modulates attention to emotional faces in patients with chronic depression.

**Methods:**

In this double-blind, randomized, controlled study, 43 patients received a single dose of oxytocin or placebo nasal spray and were tested while fulfilling a facial dot probe task. We assessed reaction times to neutral probes presented at the location of one of two faces depicting happy, angry, or neutral expressions as a prime.

**Results:**

When comparing reaction times to the congruent (prime and probe at the same location) with incongruent presentation of facial emotions, neither the placebo nor oxytocin group showed an attentional preference for emotional facial expressions in terms of a threat bias. However, oxytocin treatment did reveal two specific effects: it generally reduced the allocation of attention towards angry facial expressions, and it increased sustained attention towards happy faces, specifically under conditions of heightened awareness, i.e. trials with longer primes.

**Conclusions:**

We investigated a heterogeneous group of medicated male and female patients. We conclude that oxytocin does modulate basic factors of facial emotion processing in chronic depression. Our findings encourage further investigations assessing the therapeutic potential of oxytocin in chronic depression.

**Trial registration:**

EUDRA-CT 2010-020956-69. Date registered: 23 February 2011.

## Background

In about a third of patients with major depressive disorder, the depression becomes chronic, defined as the illness lasting longer than two years [1, 2]. However, chronic depression is not just the prolonged course of a major depressive disorder - it differs in several key psychopathological aspects from the episodic course of depression. Chronically depressed patients are characterized by a high degree of early life trauma, specifically emotional abuse and neglect [3]; they report more negative interpersonal experiences with significant others than patients with episodic depression [4] and experience worse impaired psychosocial function [1]. These features, and clinical experience from the psychological treatment of chronically depressed patients, have led to the hypothesis that patients with chronic depression are arrested in the “preoperational developmental stage” according to Piaget, which is characterized by an egocentric perspective on the world and impaired interpersonal empathy and theory of mind [5–7].

### Facial emotion recognition and attentional bias in depression

Facial emotion recognition contributes to the cognitive facet of empathy and represents a basic requisite to understanding social interactions. Numerous studies have assessed facial emotion recognition in major depression (for a review: [8]). Although the accuracy of facial emotion recognition seems to be normal, there is considerable evidence of a negative response bias towards sadness and increased vigilance and selective attention to negative emotions in depressed individuals [9]. Facial emotion recognition and attentional bias towards negative facial cues have not been assessed specifically in chronic depression. Given the high degree of psychosocial impairment that chronically depressed patients suffer, better understanding of the attentional processes for social signals in chronic depression and their neurobiological modulation might be especially relevant for clinical treatment.

### The role of attention in face processing

Socially-relevant stimuli such as emotional facial expressions normally capture our attention. Enhanced attention to emotional faces can be inferred from studies showing that emotional faces are more easily detected than neutral stimuli, as evident in visual search paradigms [10] and rapid serial presentation paradigms [11]. Thus, increased attentional preference for a given stimulus category indicates the greater motivational relevance or saliency of this specific stimulus.

A widely-used paradigm to assess attentional preference for a given category of emotional stimuli is the dot probe task [12]. In short, in the classical dot probe task, two stimuli (e.g. an emotional and neutral face) are presented simultaneously on a screen, followed by a probe, which is presented either at the target’s location (congruent) or at the distractor’s location (incongruent). Attentional preference for the target is inferred from the so-called congruency effect, i.e. if the reaction times (RTs) are significantly faster in congruent than incongruent trials.

Although several studies have confirmed a robust, threat-related bias in terms of an enhanced congruency effect in response to highly aversive/threatening stimuli in anxious populations, the congruency effect has been revealed as less consistent in healthy or subclinical samples [13]. It should be noted that most studies used verbal material or complex, emotionally-laden visual scenes as target stimuli in the dot probe task.

There is considerable evidence that depressed patients demonstrate an attentional bias towards negative information including social cues such as negative facial expressions (for a review: [9]). Initial evidence of biased attention towards emotional facial expressions arises from studies involving subclinical dysphoric samples. Dysphoric people demonstrate the tendency to avoid happy facial expressions [14], and low dysphoria is associated with an attentional bias away from angry faces [15]. There is a paucity of studies investigating patients diagnosed with major depression. Initial evidence exists of an attentional bias towards sad faces [16, 17], and angry faces [18], although other investigators did not detect biased attention to either facial stimuli in depression [19].

Some researchers have argued in favor of at least two processes underlying the congruency effect: the effect of initial allocation of attention to the salient prime in congruent trials, and the inability to reallocate attention to the probe’s location in incongruent trials. While the former might be largely due to vigilance, the latter can be referred to as adherence, or the difficulty of disengaging from the prime. Indeed, previous studies found that the congruency effect in the dot-probe task was largely due to enhanced adherence to the threat stimulus rather than increased vigilance [20, 21]. It might therefore be particularly interesting to explicitly differentiate between treatment effects on the initial allocation of attention towards a specific stimulus category and attentional adherence to the stimulus.

### Oxytocin, face processing, and social attention

The nine-amino-acid oxytocin is known to modulate social interaction in animals and humans [22, 23]. Experimental studies in humans have associated oxytocin with an enhanced ability to recognize facial emotions [24] in both healthy individuals [25–28] and in those with impaired facial emotion recognition abilities such as autism [29, 30] or schizophrenia [31, 32]. There is recent evidence that oxytocin increases covert attention to positive facial cues in healthy males [33]. Another study using a spatial cueing paradigm reported facilitated attentional disengagement from negative facial expression stimuli after the intranasal administration of oxytocin [34]. Moreover, there is evidence of positive effects on overt visual attention to the eye region in both neutral and emotional faces [35–37], suggesting that attention modulation might be a significant mediator in the beneficial effects of oxytocin on emotion recognition, although this link must still be demonstrated [27]. Finally, there are initial findings that oxytocin also has beneficial effects on affective symptoms and social cognitive functioning in patients with episodic depression [38, 39].

### Aims and hypotheses

The present study aimed to investigate whether a single dose of intranasally applied oxytocin would modulate attention towards social signals of threat (angry faces) and social approach (happy faces) in a group of patients with chronic depression. Angry faces were chosen on the basis of previous studies addressing the attention effects of oxytocin in a social context [33–35] and previous findings demonstrating a bias towards angry faces in depressed patients [18, 40]. Ellenbogen et al. demonstrated that oxytocin attenuated the attentional bias to masked angry faces in persons with high depression scores [34]. Based on findings of reduced attention to positive facial cues in depression and oxytocin’s effects on attention summarized above, we specifically hypothesized that patients receiving oxytocin prior to a facial dot probe task would reveal greater attention towards happy faces than placebo controls, as indicated by a greater congruency effect. Furthermore, we were interested in determining whether oxytocin treatment would affect initial attention allocation or adherence to the emotional target.

## Methods

### Participants

Forty-three patients with chronic depression were recruited from the in-patient and out-patient units of the Department of Psychiatry and Psychotherapy at the University Medical Center Freiburg. Patients were considered for the study if they met the criteria for chronic depression as defined by depressive symptomatology for more than two years in the course of a depressive episode or a recurrent depressive disorder (ICD-10: F32.1, F32.2, F33.1, F33.2). In addition, many patients fulfilled the criteria for dysthymia (ICD-10: F34.0) and therefore suffered from double depression (depressive episode + dysthymia). Patients were excluded from the study if they met any of the following criteria: bipolar disorder, current psychotic symptoms, alcohol or drug addiction, a clinical autism diagnosis, severe uncorrected visual impairment. Of the total sample, 41 were taking psychoactive medication: 9 patients were taking SSRI, 15 SSNRI, 7 tri-/tetracyclic antidepressants, 6 DNRI, and 4 were taking a neuroleptic or others. Patients were randomly allocated to receive a single dose of intranasally administered oxytocin (*n* = 22) or a placebo (*n* = 21) before the dot-probe task under double-blind conditions. For demographic and clinical characteristics of these groups, see Table 1. The study protocol was approved by the ethics committee of the University of Freiburg. Patients provided written-informed consent once the study procedures had been fully explained.

**Table 1:** Demographic and clinical characteristics of the study groups

		Placebo (*n* = 21)		Oxytocin (*n* = 22)	
		m	(s.d.)	m	(s.d.)
Age		47.2	(9.0)	46.7	(11.1)
Sex (m/f)		9/12		9/13	
Years in school		11.3	(2.0)	11.0	(1.5)
IQ (WST)		106.6	(9.7)	108.7	(9.6)
Depression (BDI)		28.0	(9.5)	29.1	(11.9)
Trait anxiety (STAI)		60.9	(8.3)	61.4	(8.1)
Medication (y/n)		19/2		22/0	
Illness duration (years)		12.9	(10.2)	14.8	(12.1)
Calmness	pre	13.0	(3.2)	12.5	(2.5)
	post	11.8	(2.9)	11.6	(2.4)
Wakefulness	pre	13.1	(3.5)	12.8	(4.2)
	post	10.3	(3.6)	11.0	(3.4)
Mood	pre	12.9	(3.2)	12.5	(2.5)
	post	11.8	(2.9)	11.6	(2.4)

WST, Wortschatztest; BDI, Beck Depression Inventory; STAI, State-Trait Anxiety Inventory

### Psychometric assessment

All patients completed the Beck Depression Inventory [41], the State-Trait-Anxiety Questionnaire [42] and multi-dimensional mood questionnaire [43] to assess trait depression, anxiety and changes in calmness, wakefulness and mood over the course of the experiment. The short version of the multidimensional mood questionnaire employed here comprises 12 items on three scales, measuring state calmness, wakefulness, and mood using 5-point Likert scales. The questionnaire has proven good reliability and validity and is a sensitive measure to capture state-dependent changes in calmness, wakefulness and mood [43].

### Experimental procedures

After completing demographic, clinical and mood-state questionnaires, patients self-administered 6 puffs of Syntocinon® nasal spray (Novartis, Basel, Switzerland; 3 puffs per nostril, containing 4 IU oxytocin each) or a placebo under the supervision of the experimenter. After approx. 45 min, participants completed a basic facial emotion recognition experiment before being asked to complete a dot-probe task to assess attentional preference for facial stimuli with varying valence [12]. The dot-probe thus started approx. 60 min after intranasal oxytocin administration.

**Fig. 1:**
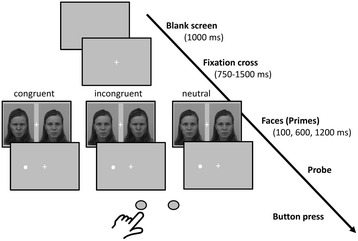
Trial structure and experimental conditions of the dot probe task. Following a fixation cross for 750–1500 ms, primes were presented simultaneously for either 100, 600, or 1200 ms. RTs were measured from the onset of the dot probe which followed the primes until the button press

As prime stimuli, happy, angry and neutral facial expressions of 40 persons (20 male/20 female) were selected from the Karolinska Directed Emotional Faces database [44]. An overview of trial structure and experimental conditions is provided in Fig. 1. A trial started with a blank screen for 1000 ms followed by a fixation cross for 750 to 1500 ms. Then a pair of angry/neutral, happy/neutral or neutral/neutral faces of the same person was presented (each 295 × 400 pixels, 12.2 × 16.5° visual angle) with an vertical offset of +/− 200 pixels (8.3° visual angle) from the screen center lasting 100, 600, or 1200 ms. Immediately following the presentation of faces, a dot probe was presented at the location of one of the faces. For the angry/neutral and happy/neutral pairs, the probe (a small black box, 10 × 10 pixels) appeared in the center of the emotional face (congruent) or the neutral face (incongruent) in half of the trials. Participants were asked to press as quickly as possible either of two buttons located in front of them on a table to indicate the probe’s location. The numbers of correct responses and RTs to the dot probes were recorded. At each presentation time (100, 600, 1200 ms), 120 trials were presented comprising 40 trials for each pairing of faces (angry-neutral, happy-neutral, neutral-neutral). In all, there were 360 trials lasting a maximum approx. 30 min. The experiment was run on a Windows PC with a 22” TFT display (40.8 × 30.6 cm; 1650 × 1050 pixel) using Presentation (Version 12.1, Neurobehavioral Systems, Albany CA, USA). Viewing distance was kept constant at 65 cm using a chin-/headrest.

### Statistical analyses

Raw RTs in erroneous trials with erroneous responses, and RTs below 300 ms and above 1500 ms were excluded from further analysis. In all, 15,480 trials were presented to the 43 participants in the study. Of these, 480 (3.1 %) were discarded from the analysis, 145 (0.9 %) were out of the time range and 335 (2.2 %) were errors. RT outliers and errors did not differ between the experimental conditions (all *p* < .10).

Raw RTs were analyzed via a four-way ANOVA comprising the factors group (oxytocin, placebo), prime duration (100, 600, 1000 ms), prime valence (happy, angry), and condition (congruent, incongruent).

Attentional bias for specific emotional facial expressions was calculated by subtracting averaged RTs to congruent trials from averaged RTs to incongruent trials within each condition (congruency effect). These bias scores were subject to a three-way ANOVA with group (oxytocin, placebo) as the between-subject factor, and presentation duration (100, 600, 1200 ms) and prime valence (angry, happy) as within-subject factors.

We also calculated prime-valence effects on initial allocation and adherence by contrasting RTs on congruent and incongruent trials with RTs on neutral control trials. Faster RT to congruent than to neutral trials reveals initial allocation of attention to the position of the salient prime location and result in positive values for the contrast ΔRT_allocation_ = RT_neutral_ – RT_congruent_. In turn, responding slower to incongruent than to neutral trials indicates adherence to the position of the salient prime and result in positive values for the contrast ΔRT_adherence_ = RT_incongruent_ – RT_neutral_. Again, both allocation and adherence scores were subjected to separate three-way ANOVAs with the factors group, presentation duration, and prime valence. Post-hoc testing employed separate follow-up two-way ANOVAs for happy and angry primes with the factors group (oxytocin, placebo) and prime duration (100, 600, 1200 ms).

Statistical significance was set to *p* < .05. In case of non-homogenous variances, the Greenhouse-Geisser correction was employed. All statistical analyses were done with SPSS 20 for Windows.

## Results

### Demographic and clinical characteristics of the study group

The two study groups were equivalent regarding age, education, gender distribution, IQ, trait anxiety, and depression. Although we noted a significant reduction in calmness, wakefulness, and mood over the course of the experiment (all *p* < .05), this effect did not substantially differ between the oxytocin and placebo group (all *p* > .10). Descriptive data are given in Table 1.

### Raw reaction time data

In the first step, we analyzed differences in raw RT data for the factors drug condition, congruency, prime duration, and emotional valence of prime. The four-way ANOVA revealed a significant main effect of prime duration, indicating overall shorter RTs on primes with longer duration (F[2,82] = 16.83; *p* < .001; η^2^_part_ = .291). No other effect attained significance, indicating that RTs were neither modulated by the emotional valence of the prime nor by the probe’s location. Furthermore, we observed no drug-condition effect (Table 2).

**Table 2:** Average raw RTs in milliseconds (s.d.) for the experimental conditions in the dot-probe task

	Placebo (*n* = 21)		Oxytocin (*n* = 22)	
	Congruent	Incongruent	Congruent	Incongruent
Happy				
100 ms	608 (96)	600 (94)	612 (99)	590 (83)
600 ms	578 (95)	575 (95)	579 (93)	572 (95)
1200 ms	551 (93)	554 (81)	577 (95)	578 (90)
Angry				
100 ms	598 (117)	585 (107)	602 (82)	609 (100)
600 ms	568 (89)	562 (102)	580 (83)	579 (85)
1200 ms	550 (93)	555 (92)	581 (97)	568 (91)
Neutral				
100 ms	586 (92)		599 (86)	
600 ms	565 (85)		570 (80)	
1200 ms	555 (85)		559 (83)	

### Attentional bias – congruency effect

To analyze the modulation of attentional preference for happy vs. angry faces at different prime durations, we analyzed attentional bias scores (difference between RTs to congruent vs. incongruent trials). In the three-way ANOVA testing for effects of prime duration, prime valence and drug condition, neither any of the main effects nor any of the interactions attained significance. We thus detected no overall modulation of attentional preference for emotional facial expressions in terms of a congruency effect (Table 3).

**Table 3:** Attentional bias scores (in ms) for the different experimental conditions in the dot-probe task

	Placebo (*n* = 21)		Oxytocin (*n* = 22)	
	m	(s.d.)	m	(s.d.)
Happy				
100 ms	−7.7	(38.8)	−21.4	(55.5)
600 ms	−2.7	(46.8)	−7.9	(43.8)
1200 ms	3.0	(47.4)	0.9	(35.5)
Angry				
100 ms	−13.3	(44.3)	8.3	(39.7)
600 ms	−6.2	(41.9)	−0.6	(38.6)
1200 ms	4.0	(40.7)	−11.6	(52.7)

### Allocation vs. adherence

**Fig. 2:**
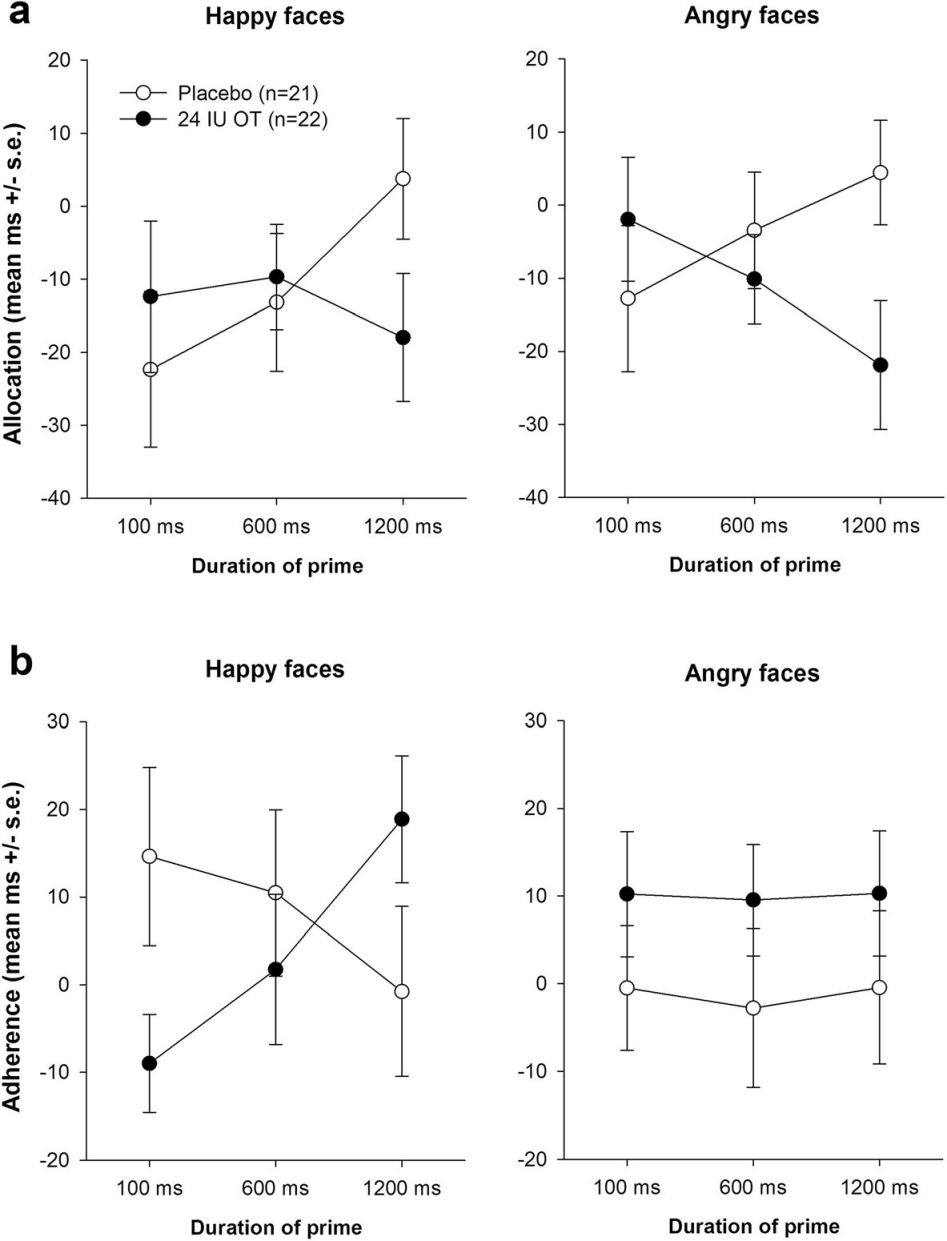
Effects of oxytocin on the initial allocation of attention to the emotional prime and adherence on the emotional prime face as a function of presentation duration and emotional valence. **a** Allocation scores are calculated by subtracting the average RT to congruent trials from the average RT to neutral trials. Thus, negative values represent longer RTs to probes at the congruent location of the emotional face compared to neutral trials. **b** Adherence scores are calculated by subtracting the average RT to neutral trials from the average RT to incongruent trials. Thus, positive scores indicate sustained attention to the emotional prime

To further investigate effects on the initial allocation of attention towards the salient emotional prime and effects on adherence on salient primes, we performed two separate three-way ANOVAs. In the allocation scores, we noted significant interaction between prime duration and drug condition (F[2,82] = 3.54; *p* = .033; η^2^_part_ = .080): For both happy and angry faces, the placebo group displayed a greater initial allocation of attention with increasing duration of the salient emotional prime, while the oxytocin group demonstrated decreased initial allocation towards the emotional prime with increasing prime duration (Fig. 2a). In the follow-up two-way ANOVA with the factors group and prime duration for angry primes, we noted a significant group by prime duration interaction (F[2,82] = 3.20; *p* = .046, η^2^_part_ = .073), while there was no such effect in the follow-up ANOVA for happy faces (F[2,82] = 1.87; *p* = .161), suggesting reduced allocation of attention with increasing duration of the angry prime after oxytocin treatment.

For adherence scores, i.e. slower attentional disengagement from the salient prime, we identified a significant interaction between prime valence and drug condition (F[2,82] = 6.64; *p* = .014; η^2^_part_ = .139) and a marginally significant interaction of prime valence, prime duration and drug condition (F[2,82] = 2.57; *p* = .083; η^2^_part_ = .059). While the oxytocin group demonstrated greater adherence to happy faces with increasing prime duration than the placebo group, there was no such modulation for angry faces (Fig. 2b). Follow-up two-way ANOVAs with the factors group and prime duration confirmed the differential effect of oxytocin with happy and angry faces: for happy faces a significant prime duration by drug interaction was observed, (F[2,82] = 3.21; *p* = .046; η^2^_part_ = .073), while there was no significant effect in association with the angry faces (F[2,82] = 0.08; *p* = .992).

## Discussion

This study provides initial evidence for an oxytocin-induced modulation of attention towards positive and negative social signals in chronic depression. Although no congruency effect was observed in the present study, and the placebo group demonstrated no negativity or threat-bias, oxytocin did reduce the initial allocation of attention towards angry faces, and it increased sustained attention towards happy faces, specifically under conditions of heightened awareness, i.e. during trials with longer prime duration.

The lack of an overall attentional threat bias concurs with some previous studies and might be attributable to this measure’s limited sensitivity, especially when attention allocation and adherence reveal reversed effects and thus cancel each other out [21]. Inspecting the pattern of results (Fig. 2), this might be the case in the present study, since oxytocin exhibits opposing effects in conjunction with allocation and adherence of attention depending on the emotion presented.

Increased adherence or an attenuated tendency to disengage from the incongruent salient prime seems to be the primary factor underlying the congruency effect in investigations with anxious patients [20, 21]. Although there are very few studies addressing visual attention to specific social cues in depression and (to our knowledge) none examining chronic depression, there is initial evidence that patients suffering from major depression display reduced attention to positive social cues (happy faces) and increased attention to negative (sad and angry) ones [17, 18, 45]. In this study, we report increased adherence to positive social cues after oxytocin treatment, a finding in line with an earlier study using eye-tracking in healthy participants [35]. This effect might in part compensate for the attentional negativity bias previously observed, and might thus demonstrate beneficial effects on social interaction in chronic depression.

Furthermore, our data suggest decreased initial allocation for negative facial expressions in the group receiving oxytocin compared to placebo. Notably, a recent study demonstrated that selective attention toward angry faces is a significant predictor for the recurrence of depression [40]. In light of these results, an oxytocin-induced shift of attention from negative to positive social cues might exert protective effects in the course of chronic illness or recurrent depression. However, the oxytocin group’s negative allocation-score values regarding increased prime durations indicate prolonged reaction times to congruent probes, although the attentional focus is presumably primed to the correct location. One plausible explanation for this paradoxical priming effect is the possibility that processing the facial stimulus interferes with the response to the probe. Negative allocation scores might therefore also indicate increased adherence to the salient congruent prime, and could reveal increased attention to the prime faces rather than a reduced allocation of attention.

The following limitations should be acknowledged. Although we observed significant modulations in attentional processes via the experimental intranasal administration of a single dose of oxytocin in a group of patients with chronic depression, interpretations regarding its clinical application should be made cautiously. First, the overall effects observed in this study were small and thus require replication. Attention to social stimuli is obviously just one of many other factors contributing to real-life social interactions. Follow-up studies are thus needed to evaluate the relative contribution of attentional processes to social functioning. Second, we investigated a heterogeneous group of male and female patients of whom almost all were taking psychoactive medication. It would therefore be an interesting topic for future studies to elucidate the role of sex and gonadal steroids in the effects we observed, as well as any interactions with serotonergic or noradrenergic medication. Third, long-term randomized controlled clinical trials are needed to evaluate the usefulness of intranasal oxytocin as an add-on to well-established cognitive behavioral treatment programs or pharmacotherapy in chronic depression. Previous studies suggest altered facial emotion perception related to personality disorders [46, 47]. In the present study none of the patients had a clinical diagnosis of BPD or any other personality disorder. However, lacking a structured interview, we cannot rule out that some patients met some of those criteria.

## Conclusions

To summarize: there is increasing evidence of a role played by oxytocin in human social cognition and interaction [22, 23]. In addition, recent studies suggest that individuals with impaired social cognitive abilities might benefit from increased availability of central nervous oxytocin [29, 31, 48–52]. Previous studies with depressed patients have reported positive effects of oxytocin on affective symptoms and social cognition [38, 39]. Here we report increased attention to emotional faces in chronic depression using an experimental paradigm specifically tailored to disentangle the effects on allocation and adherence of visual attention for positive and negative social cues. In particular, our results suggest decreased allocation of attention to aversive social signals, and increased adherence to positive social signals after oxytocin treatment in patients with chronic depression. Given the psychopathological differences between episodic and chronic depression, the present study might be considered a point of departure for deeper research into the role of oxytocin in chronic depression – a disorder characterized by pronounced and disabling difficulties in social cognition and functioning [1].

### Ethical approval and consent to participate

The ethics committee at the University of Freiburg approved the study (EK.-Nr. 307/10) and participants gave written-informed consent before participation.

### Consent to publish

Consent to publish is not required.

### Availability of data and materials

By contact with corresponding author.
